# Supplementary material for: Clinical Implications of Prominent Cortical Vessels on Susceptibility-Weighted Imaging in Acute Ischemic Stroke Patients Treated with Recanalization Therapy
Source: Brain Sci. 2022 Jan 29;12(2):184. doi: 10.3390/brainsci12020184 (PMC8869791; doi:10.3390/brainsci12020184)
Supplement: Supplementary file 1 [file brainsci-12-00184-s001.zip › brainsci-1565097-supplementary.pdf]

Supplementary materials

Table S1. Clinical and imaging characteristics according to functional outcome at 3 months.

	Patients, No. (%)		
	Good functional outcome 3-month mRS 0-2 (n=36)	Poor outcome 3-month mRS 3-6 (n=64)	p-value
Demographics and clinical characteristics			
Age, mean (SD), years	66 (14)	73 (11)	0.03 [a]
Male sex	23 (63.9)	31 (48.4)	0.14 [b]
Baseline SBP, median (IQR), mmHg	140 (130, 150)	144 (130, 164)	0.25 [c]
Baseline glucose level, median (IQR) mg/dl	116 (106, 142)	139 (119, 172)	<0.01 [c]
Baseline NIHSS score, median (IQR)	13 (9, 17)	16 (14, 19)	<0.01 [c]
Pre-stroke mRS			0.07 [d]
0	35 (97.2)	55 (85.9)	
1	1 (2.8)	7 (10.9)	
2	0	2 (3.1)	
TOAST classification			0.22 [d]
Large artery atherosclerosis	10 (27.8)	9 (14.1)	
Cardioembolism	20 (55.6)	43 (67.2)	
Other determined or undetermined	6 (16.7)	12 (18.8)	
History of stroke	3 (8.3)	17 (26.6)	0.04 [e]
Hypertension	18 (50.0)	52 (81.3)	<0.01 [b]
Diabetes mellitus	6 (16.7)	18 (28.1)	0.20 [b]
Dyslipidemia	5 (13.9)	9 (14.1)	0.98 [b]

Atrial fibrillation	18 (50.0)	39 (60.9)	0.29 [b]
Current smoker	9 (25.0)	13 (20.3)	0.59 [b]
Pre-stroke medication			
Antiplatelet or anticoagulants agents	10 (27.8)	31 (48.4)	0.04 [b]
Statin	4 (11.1)	13 (20.3)	0.28 [e]
Reperfusion therapy type			0.61 [d]
IV thrombolysis	7 (19.4)	3 (4.7)	
Endovascular treatment	6 (16.7)	25 (39.1)	
Combined therapy	23 (63.9)	36 (56.3)	
Site of occlusion			0.01 [d]
Middle cerebral artery			
M1	29 (80.6)	30 (46.9)	
M2	1 (2.8)	3 (4.7)	
Internal carotid artery	6 (16.7)	31 (48.4)	
ASPECTS-SWI, median (IQR)	8 (6, 9)	5 (3, 7)	<0.01 [c]
PCV-SWI (ASPECT-SWI, 0-7)	17 (25.4)	50 (74.6)	<0.01 [b]
Collateral score-mCTA, median (IQR)	4 (3, 4)	3 (2, 4)	<0.01 [c]
Good collateral status (CC-mCTA, 4-5)	22 (61.1)	22 (34.4)	0.01 [b]
Successful recanalization	30 (83.3)	33 (51.6)	<0.01 [b]
Any intracerebral hemorrhage	5 (13.9)	22 (34.4)	0.03 [b]

Abbreviations: SD, standard deviation; IQR, interquartile range; mRS, modified Rankin scale; NIHSS, National Institute of Health Stroke Scale; TOAST, Trial of ORG 10172 in Acute Stroke Treatment; LAA, large artery atherosclerosis; CE, cardioembolism; OE, other etiology; mCTA, multiphase CTA; ASPECTS, Alberta Stroke Program Early CT Score; eTICI, expanded Thrombolysis in Cerebral Infarction; mRS, modified Rankin Scale

[a] p-value by student’s t test

[b] p-value by chi-square test

[c] p-value by Mann-Whitney U test

[d] p-value by Cochran-Mantel-Haenszel shift test

[e] p-value by Fisher's exact test

Table S2. Clinical and imaging characteristics according to successful recanalization (eTICI 2b-3).

	Patients, No. (%)		p-value
	eTICI 2b-3 (n=62)	eTICI 0-2a (n=38)	
Demographics and clinical characteristics			
Age, mean (SD), years	70 (13)	70 (13)	0.85 [a]
Male sex	34 (54.0)	20 (54.1)	0.99 [b]
Baseline SBP, median (IQR), mmHg	140 (130, 160)	140 (130, 160)	0.93 [c]
Baseline glucose level, median (IQR) mg/dl	129 (111, 155)	131 (119, 180)	0.18 [c]
Baseline NIHSS score, median (IQR)	16 (12, 18)	15 (11, 19)	0.85 [c]
Pre-stroke mRS			0.19 [d]
0	56 (87.3)	35 (94.6)	
1	6 (9.5)	2 (9.5)	
2	2 (3.2)	0	
TOAST classification			0.58 [d]
Large artery atherosclerosis	9 (14.3)	10 (27.0)	
Cardioembolism	44 (69.8)	19 (51.4)	
Other determined or undetermined	10 (15.9)	8 (21.6)	
History of stroke	18 (28.6)	2 (5.4)	0.01 [e]

Hypertension	42 (66.7)	28 (75.7)	0.34 [b]
Diabetes mellitus	16 (25.4)	8 (21.6)	0.67 [b]
Dyslipidemia	9 (14.3)	5 (13.5)	0.91 [b]
Atrial fibrillation	39 (61.9)	18 (48.6)	0.19 [b]
Current smoker	15 (23.8)	7 (18.9)	0.57 [b]
Pre-stroke medication			
Antiplatelet or anticoagulants agents	28 (44.4)	13 (35.1)	0.36 [b]
Statin	13 (20.6)	4 (10.8)	0.28 [e]
Reperfusion therapy type			0.95 [d]
IV thrombolysis	9 (14.3)	6 (16.2)	
Endovascular treatment	20 (31.7)	10 (27.0)	
Combined therapy	34 (54.0)	21 (56.8)	
Site of occlusion			0.16 [d]
Middle cerebral artery			
M1	38 (60.3)	21 (56.8)	
M2	4 (6.3)	0	
Internal carotid artery	21 (33.3)	16 (43.2)	
ASPECTS-SWI, median (IQR)	7 (4, 8)	5 (4, 7)	<0.01 [c]
PCV-SWI (ASPECT-SWI, 0-7)	36 (58.1)	31 (81.6)	0.02 [c]
Collateral score-mCTA, median (IQR)	3 (3, 4)	3 (2, 4)	0.06 [b]
Good collateral status (CC-mCTA, 4-5)	30 (47.6)	14 (37.8)	0.34 [b]

Abbreviations: eTICI, expanded Thrombolysis in Cerebral Infarction; SD, standard deviation; IQR, interquartile range; mRS, modified Rankin scale; NIHSS, National Institute of Health Stroke Scale; TOAST, Trial of ORG 10172 in Acute Stroke Treatment; LAA, large artery atherosclerosis; CE, cardioembolism; OE,

other etiology; mCTA, multiphase CTA; mRS, modified Rankin Scale; ASPECTS, Alberta Stroke Program Early CT Score; PCV-SWI, prominent cortical vessel-susceptibility weighted image; CC-mCTA, collateral status score-multiphase CT angiography

[a] p-value by student's t test

[b] p-value by chi-square test

[c] p-value by Mann-Whitney U test

[d] p-value by Cochran-Mantel-Haenszel shift test

[e] p-value by Fisher's exact test

Table S3. The factors associated with successful recanalization (eTICI 2b or 3) using logistic regression analyses.

	Unadjusted OR		Adjusted OR	
	OR (95% CI)	p-value	OR (95% CI)	p-value
Age	1.00 (0.97, 1.04)	0.85	1.01 (0.98, 1.05)	0.48
Sex (male)	0.99 (0.44, 2.25)	0.99	1.04 (0.41, 2.63)	0.94
History of stroke	7.00 (1.52, 32.2)	0.01	5.95 (1.53, 23.09)	0.01
PCV-SWI	0.31 (0.12, 0.82)	0.02	0.23 (0.08, 0.65)	<0.01

Abbreviations: OR, odds ratio; CI, confidence interval; NIHSS, National Institute of Health Stroke Scale; PCV-SWI, prominent cortical vessel- susceptibility weighted image

Adjusted for age, sex, history of prior stroke, and prominent cortical vessels (PCV-SWI 0-2)

Table S4. Clinical and imaging characteristics according to any intracerebral hemorrhage.

	Patients, No. (%)		
	Any ICH (+) (n=27)	Any ICH (-) (n=73)	p-value
Demographics and clinical characteristics			
Age, mean (SD), years	69.4 (11.9)	70.4 (13.2)	0.74 [a]
Male sex	15 (55.6)	39 (53.4)	0.85 [b]
Baseline SBP, median (IQR), mmHg	140 (137, 170)	140 (130, 158)	0.53 [c]
Baseline glucose level, median (IQR) mg/dl	147(120, 173)	124 (110, 153)	0.02 [c]
Baseline NIHSS score, median (IQR)	16 (14, 18)	15 (11, 18)	0.29 [c]
Pre-stroke mRS			0.66 [d]
0	24 (88.9)	66 (90.4)	
1	2 (7.4)	6(8.2)	
2	1 (3.7)	1 (1.4)	
TOAST classification			0.12 [d]
Large artery atherosclerosis	1 (3.7)	18 (24.7)	
Cardioembolism	21 (77.8)	42 (57.5)	
Other determined or undetermined	5 (18.5)	13 (17.8)	
Stroke history	8 (29.6)	12 (16.4)	0.14 [b]
Hypertension	21 (77.8)	49 (67.1)	0.30 [b]
Diabetes mellitus	10 (37.0)	14 (28.1)	0.06 [b]
Dyslipidemia	5 (18.5)	9 (12.3)	0.43 [b]
Atrial fibrillation	19 (70.4)	38 (52.1)	0.10 [b]
Current smoker	6 (22.2)	16 (21.9)	0.97 [b]
Pre-stroke medication			

Antiplatelet or anticoagulants agents	14 (51.9)	27 (37.0)	0.18 [b]
Statin	4 (14.8)	13 (17.8)	1.00 [e]
Reperfusion therapy type			0.50 [d]
IV thrombolysis	1 (3.7)	14 (19.2)	
Endovascular treatment	12 (44.4)	18 (24.7)	
Combined therapy	14 (51.9)	41 (56.2)	
Site of occlusion			0.66 [d]
Middle cerebral artery			
M1	1 (3.7)	3 (4.1)	
M2	15 (55.6)	44 (60.3)	
Internal carotid artery	11 (40.7)	26 (35.6)	
ASPECTS-SWI, median (IQR)	5 (2, 8)	6 (4, 8)	0.26 [c]
PCV-SWI (ASPECT-SWI, 0-7)	19 (70.4)	48 (65.8)	0.66 [b]
Collateral score-mCTA, median (IQR)	3 (2, 4)	3 (2, 4)	0.50 [c]
Good collateral status (CC-mCTA, 4-5)	10 (37.0)	34 (46.6)	0.39 [b]

Abbreviations: ICH, intracerebral hemorrhage; SD, standard deviation; IQR, interquartile range; mRS, modified Rankin scale; NIHSS, National Institute of Health Stroke Scale; TOAST, Trial of ORG 10172 in Acute Stroke Treatment; LAA, large artery atherosclerosis; CE, cardioembolism; OE, other etiology; ASPECTS, Alberta Stroke Program Early CT Score; mCTA, multiphase CTA; eTICI, expanded Thrombolysis in Cerebral Infarction; mRS, modified Rankin Scale

[a] p-value by student's t test

[b] p-value by chi-square test

[c] p-value by Mann-Whitney U test

[d] p-value by Cochran-Mantel-Haenszel shift test

[e] p-value by Fisher's exact test

Table S5. The factors associated with any intracerebral hemorrhage using logistic regression analyses.

	Unadjusted OR		Adjusted OR	
	OR (95% CI)	p-value	OR (95% CI)	p-value
Age	0.99 (0.96, 1.03)	0.85	0.99 (0.96, 1.03)	0.77
Sex (male)	1.09 (0.45, 2.65)	0.14	1.11 (0.43, 2.90)	0.83
Initial glucose level, mg/dl	1.01 (0.99, 1.01)	0.21	1.01 (0.99, 1.02)	0.21
PCV-SWI	1.24 (0.48, 3.22)	0.66	1.33 (0.50, 3.55)	0.57

Abbreviations: OR, odds ratio; CI, confidence interval; NIHSS, National Institute of Health Stroke Scale; PCV-SWI, prominent cortical vessel- susceptibility weighted image

Adjusted for age, sex, glucose level at admission, and prominent cortical vessels (PCV-SWI 0-2)

Table S6. Predictive ability of each imaging modality to discriminate outcomes in patients with endovascular treatment using multivariable logistic regression analysis, receiver operating curve analysis, AIC, and BIC.

	Imaging modality	Adjusted OR (95% CI)	p-value	*C* statistic	AIC	BIC
Good functional	ASPECT-SWI (0-10), increase per 1 score	1.46 (1.13, 1.89)	<0.01	0.89	83.4	105
outcome	PCV-SWI (≤7 versus >7)	0.24 (0.06, 0.87)	0.03	0.86	88.9	111
(3-month mRS 0-2) [a]	CC-mCTA (0-5), increase per 1 score	2.07 (1.18, 3.63)	0.01	0.88	85.9	108
	Good CC-mCTA (≥4 versus <4)	1.52 (0.47, 4.98)	0.49	0.80	93.4	115
Successful	ASPECT-SWI (0-10), increase per 1 score	1.30 (1.09, 1.55)	<0.01	0.70	113	125
recanalization	PCV-SWI (≤7 versus >7)	0.18 (0.05, 0.59)	<0.01	0.70	112	124
(eTICI 2b-3) [c]	CC-mCTA (0-5), increase per 1 score	1.52 (1.03, 2.23)	0.04	0.71	110	122
	Good CC-mCTA (≥4 versus <4)	1.64 (0.62, 4.38)	0.32	0.66	114	126

Any intracerebral hemorrhage [d]	ASPECT-SWI (0-10), increase per 1 score	0.97 (0.82, 1.15)	0.69	0.66	112	124
	PCV-SWI (≤7 versus >7)	1.08 (0.39, 3.01)	0.89	0.67	112	124
	CC-mCTA (0-5), increase per 1 score	1.05 (0.71, 1.55)	0.82	0.68	112	124
	Good CC-mCTA (≥4 versus <4)	0.92 (0.33, 2.55)	0.88	0.67	112	124

Abbreviations: OR, odds ratio; CI, confidence interval; AIC< Akaike information criterion; BIC, Bayesian information criterion; mRS, modified Rankin scale; NIHSS, National Institute of Health Stroke Scale; PCV-SWI, prominent cortical vessel- susceptibility weighted image; CC-mCTA, collateral status score-multiphase CT angiography

[a.] Adjusted for age, sex, baseline NIHSS score, baseline glucose level, history of hypertension, history of stroke, prior antiplatelet or anticoagulant use, and each imaging modality

[b.] Adjusted for age, sex, history of hypertension, and each imaging modality

[c.] Adjusted for age, sex, admission glucose level, and each imaging modality

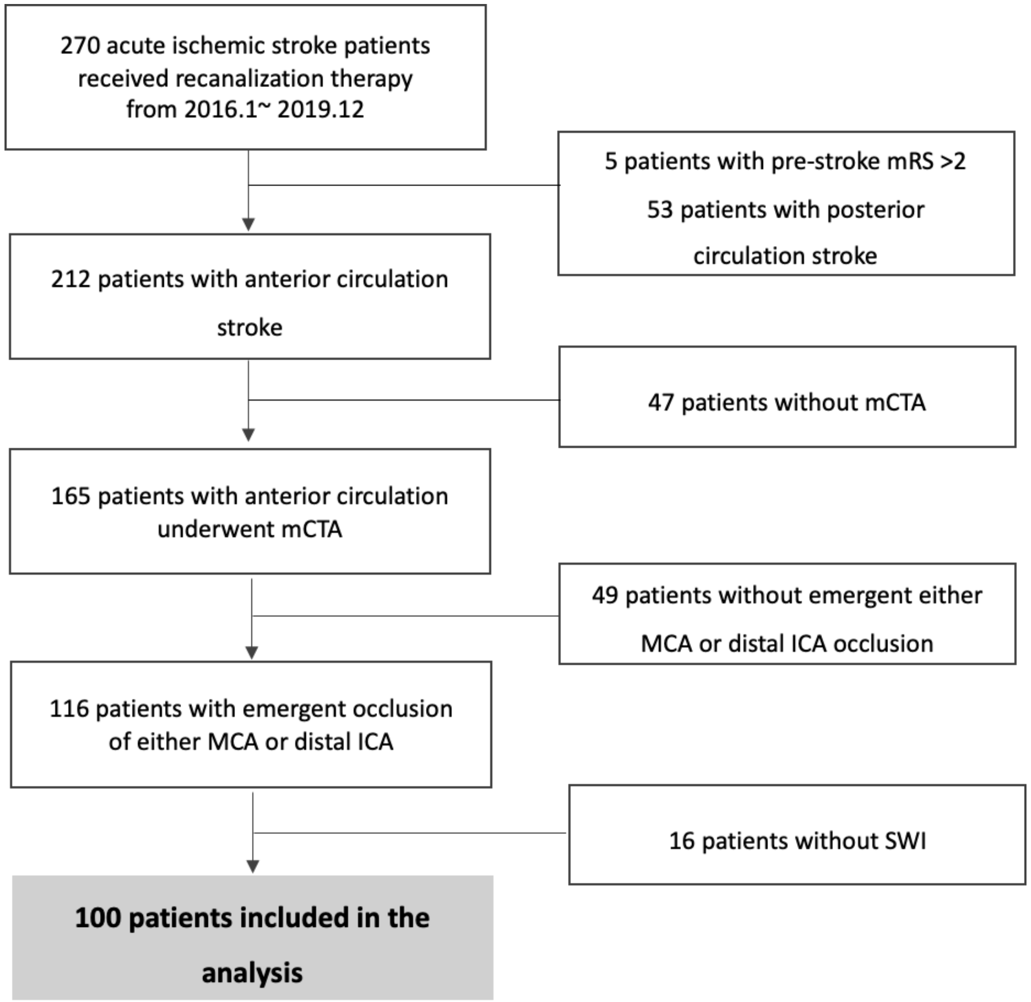

Figure S1 Study population

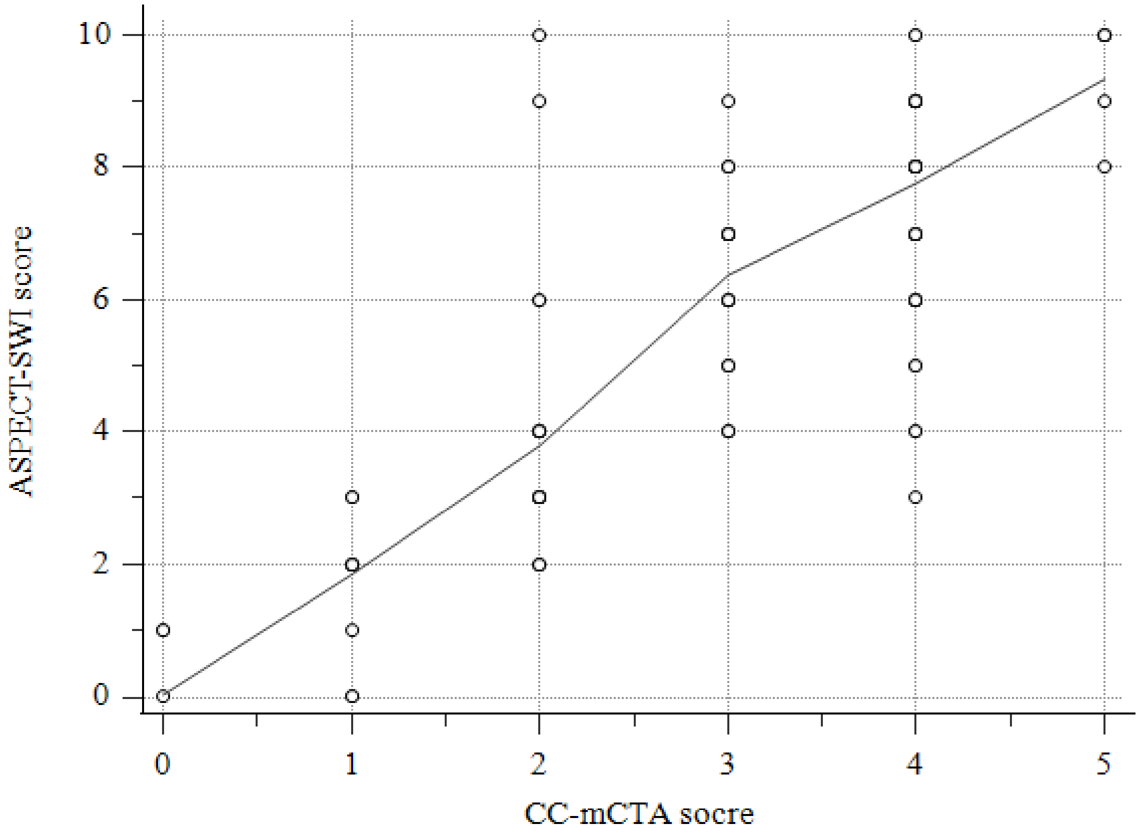

Figure S2. catter plot of the correlation between the prominent cortical vessels on SWI (PCV-SWI) and collateral status on multiphase CT angiography (CC-mCTA).

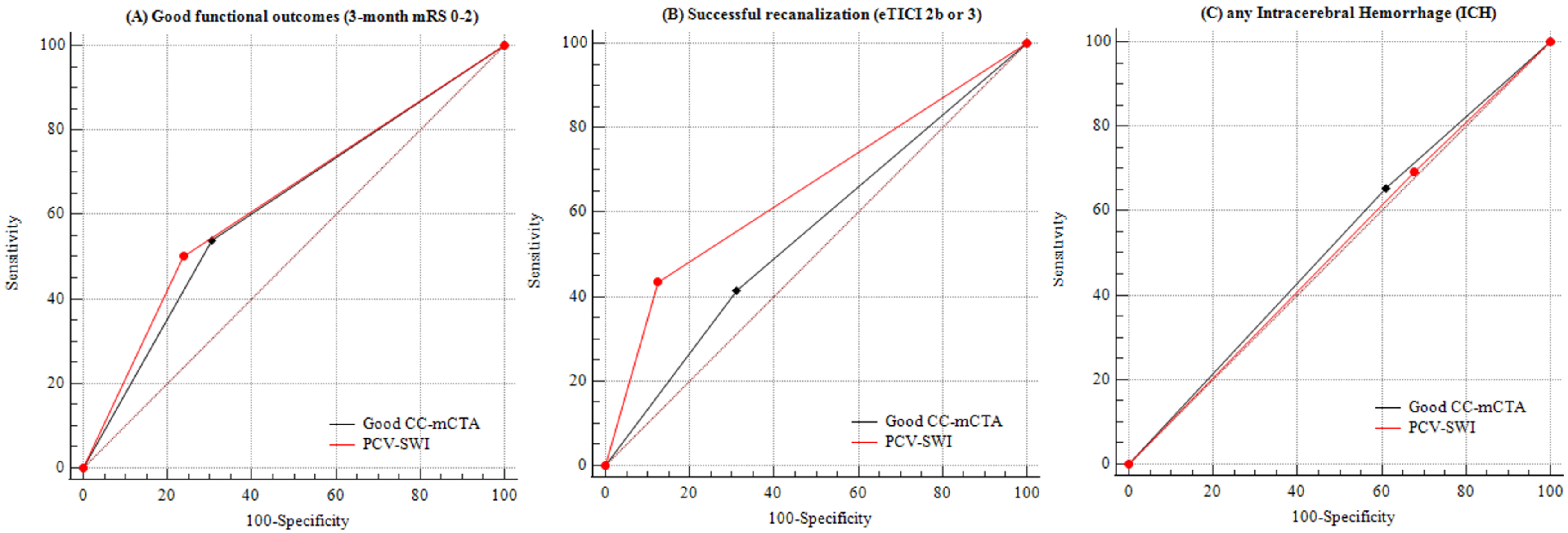

Figure S3. Comparison of AUCs between PCV-SWI and good CC-mCTA in patients with endovascular therapy.
